# Disrupted Structural and Functional Connectivity in Prefrontal-Hippocampus Circuitry in First-Episode Medication-Naïve Adolescent Depression

**DOI:** 10.1371/journal.pone.0148345

**Published:** 2016-02-10

**Authors:** Haiyang Geng, Feng Wu, Lingtao Kong, Yanqing Tang, Qian Zhou, Miao Chang, Yifang Zhou, Xiaowei Jiang, Songbai Li, Fei Wang

**Affiliations:** 1 Department of Radiology, The First Affiliated Hospital of China Medical University, Shenyang, 110001, Liaoning, PR China; 2 Department of Psychiatry, The First Affiliated Hospital of China Medical University, Shenyang, 110001, Liaoning, PR China; 3 Department of Gerontology, The First Affiliated Hospital of China Medical University, Shenyang, 110001, Liaoning, PR China; 4 Department of Psychiatry, Yale University School of Medicine, New Haven, CT, 06511, United States of America; Beijing Normal University, CHINA

## Abstract

**Background:**

Evidence implicates abnormalities in prefrontal-hippocampus neural circuitry in major depressive disorder (MDD). This study investigates the potential disruptions in prefrontal-hippocampus structural and functional connectivity, as well as their relationship in first-episode medication-naïve adolescents with MDD in order to investigate the early stage of the illness without confounds of illness course and medication exposure.

**Methods:**

Diffusion tensor imaging and resting-state functional magnetic resonance imaging (rs-fMRI) data were acquired from 26 first-episode medication-naïve MDD adolescents and 31 healthy controls (HC). Fractional anisotropy (FA) values of the fornix and the prefrontal-hippocampus functional connectivity was compared between MDD and HC groups. The correlation between the FA value of fornix and the strength of the functional connectivity in the prefrontal cortex (PFC) region showing significant differences between the two groups was identified.

**Results:**

Compared with the HC group, adolescent MDD group had significant lower FA values in the fornix, as well as decreased functional connectivity in four PFC regions. Significant negative correlations were observed between fornix FA values and functional connectivity from hippocampus to PFC within the HC group. There was no significant correlation between the fornix FA and the strength of functional connectivity within the adolescent MDD group.

**Conclusions:**

First-episode medication-naïve adolescent MDD showed decreased structural and functional connectivity as well as deficits of the association between structural and functional connectivity shown in HC in the PFC-hippocampus neural circuitry. These findings suggest that abnormal PFC-hippocampus neural circuitry may present in the early onset of MDD and play an important role in the neuropathophysiology of MDD.

## Introduction

Adolescent major depressive disorder (MDD) is associated with increased risk of suicide, behavior disorders and long-term functional impairment into adulthood[1,2]. MDD in adolescents is characterized by heterogeneous symptoms and higher comorbidity rates with substance abuse, anxiety disorders and personality disorders than those in adults[3,4], implicating differentiated pathophysiological mechanisms between adolescent MDD and adult MDD. As the substantial brain maturation taken place during adolescence[5] and the minimized confounds of other illness course and medication exposure, investigation of the neurobiology of adolescent MDD may reveal the role of abnormal developmental process leading to MDD and help us to further understand the pathophysiology of MDD.

Dysregulation of frontal-limbic neural circuits including dorsolateral prefrontal cortex (DLPFC), ventral prefrontal cortex (VPFC), anterior cingulate cortex (ACC), hippocampus and amygdala is implicated to the pathophysiology of MDD[6,7,8,9,10,11]. Convergent evidence from magnetic resonance imaging (MRI) studies reported grey matter structural deficits in these regions within MDD adults[12,13,14,15,16], as well as adolescents[17,18]. As white matter fiber tracts structural connecting brain regions into neural circuits, diffusion tensor imaging (DTI), an MRI technique detecting white matter microstructure integrity in vivo, has been used to explore white matter abnormalities relevant to the neurobiology of MDD. Numerous DTI studies have shown white matter abnormalities in prefrontal, limbic and thalamic projection fibers[19,20] and corpus callosum[21] within adult MDD. Our previous DTI study also demonstrated abnormalities in the superior longitudinal fasciculus within DLPFC as well as middle frontal white matter in the early stage of MDD[22], suggesting that disrupted structural connectivity of frontal-limbic neural circuits may play an important role in the pathophysiology of MDD. Until now, only a few studies reported white matter abnormalities in adolescent MDD[23,24,25]. However, results are inconsistent in these studies, probably due to differences in numbers of episode, comorbidity with other mental disorders and medication exposures. Therefore white matter abnormalities within frontal-limbic neural circuits can be further investigated in adolescent MDD.

The hippocampus, the key region of the limbic system involved in the process of memory formation, stress and emotional regulation[26], has been implicated in the pathophysiology of MDD[27]. Functional magnetic resonance imaging studies have demonstrated aberrant hippocampal activation in adult MDD[28,29,30]. Resting state functional magnetic resonance imaging (rs-fMRI), which can reflect individuals’ natural mental state at rest, has been used to examine neural connections within neural circuitry in vivo[31]. In rs-fMRI, resting state functional connectivity (rsFC) is indicated by the temporal correlations between low-frequency blood oxygen level-dependent (BOLD) signal fluctuations in spatially separated regions[32]. Studies using this method have reported abnormal functional connectivity of prefrontal-hippocampus circuitry in adult MDD[33,34]. Recently, Peng et al reported decreased rsFC between hippocampus and insula among medication-naïve adult MDD patients[8]. Wang et al reported abnormal functional connectivity networks of hippocampal subreigons in late-life depression[35]. These findings suggest that aberrant hippocampal rsFC networks may be involved in the neuropathophysiology of MDD. Until now, no prior studies investigated hippocampal rsFC in adolescent MDD.

The relationship between structural and functional connectivity in MDD is not fully explored. Only a prior study reported that structural abnormalities are associated with increased functional connectivity between ACC and amygdala in adult MDD[36]. In the current study, we combined DTI and rs-fMRI to examine the structural and functional connectivity within prefrontal-hippocampus neural circuitry in first-episode medication-naïve adolescents with MDD. We hypothesized that in adolescent MDD, there would be altered structural and functional connectivity between hippocampus and the prefrontal cortex (PFC), as well as an association between the structural and functional connectivity in this circuitry.

## Methods

### Subjects

Twenty-six medication-naïve adolescents with MDD were recruited from the outpatient clinic at the Department of Psychiatry, Frist Affiliated Hospital of China Medical University and Mental Health Center of Shenyang. All MDD patients were diagnosed by two trained psychiatrists using the Schedule for Affective Disorders and Schizophrenia for School-Age Children (KSADS-PL) and met the following inclusion criteria: fulfilling KSADS-PL criteria; first depressive episode; aged 13 to 17; no comorbid diagnosis of psychosis, bipolar disorder; and no history of psychotropic medication, electroconvulsive therapy or psychotherapy. Severity of depression was assessed through the 17-item Hamilton Depression Rating Scale (HAMD-17).

We also recruited thirty-one healthy controls (HC) matched for gender, age and education by advertisements. Healthy adolescents were excluded from the study if they had any Axis I psychiatric disorder. Subjects were also excluded if they had any family history of mood or psychotic disorders in first- or second-degree relatives.

Additional exclusion criteria for all participants included the following: contraindications for MRI; history of head injury or neurological disorder; history of substance abuse or dependence; any concomitant medical disorder. All participants were right-handed and were scanned within 24h of initial contact. Participants gave written informed assent, and their parent/legal guardian provided written informed consent after receiving a detailed description of the study. The study was approved by the Institutional Review Board of the China Medical University.

### MRI data acquisition

Magnetic resonance imaging was performed on a GE Signa HDX 3.0T MRI scanner with a standard head coil at the First Affiliated Hospital of China Medical University, Shenyang, China. Diffusion tensor imaging was acquired using spin-echo planar imaging sequence aligned to the anterior commissure-posterior commissure (AC-PC) plane. The diffusion sensitizing gradients were applied along 25 non-collinear directions (b = 1000s/mm^2^), together with an axial acquisition without diffusion weighting (b = 0). The scan parameters were as follows: TR = 17000ms, TE = 85.4ms, FOV = 24cm×24cm, imaging matrix = 120×120, 65 contiguous axial slices of 2mm without gap. Functional image was collected using a gradient-echo planar imaging(EPI) sequence, parallel to the AC–PC plane with the following scan parameters: TR = 2000ms, TE = 30ms, flip angle = 90°, FOV = 24 × 24cm, matrix = 64 × 64. Thirty-five axial slices were collected with 3mm thickness without gap. Participants were instructed to rest with their eyes closed but remain awake during scanning. No participant reported falling asleep during the scan when routinely asked immediately after scanning. The high resolution structural image was acquired using a three-dimensional fast spoiled gradient-echo T1-weighted sequence: TR = 7.1ms,TE = 3.2ms, FOV = 24cm×24cm, matrix = 240×240, slice thickness = 1.0mm without gap,176 slices.

### MRI data processing and analysis

#### DTI data processing

DTI data were processed by PANDA software[37] (a Pipeline for Analysing braiN Diffusion images 1.2.3 http://www.nitrc.org/projects/panda/), which synthesizes procedures in FSL (http://fsl.fmrib.ox.ac.uk/fsl), diffusion toolkit (http://www.nmr.mgh.harvard.edu/~rpwang/dtk), and MRIcron (http://www.mccauslandcenter.sc.edu/mricro/mricron). Steps are as follows: converting DICOM files into NIfTI images, estimating the brain mask, cropping images, correcting for the eddy-current effect, averaging acquisitions, calculating DTI metrics, finally, producing diffusion metrics that are ready for statistical analysis. The individual images of the diffusion metrics were transformed from native space to a standard Montreal Neurological Institute (MNI) space via spatial normalization (voxel size 1mm×1mm×1mm). The ICBM-DTI-81 WM labels atlas in the standard space allow for parcellation of the entire white matter into multiple regions of interest (ROI) [38]. PANDA calculates the regional diffusion metrics by averaging the values within each region of the white matter atlases. In our study, we selected Fornix as the ROI.

#### FMRI data processing

The fMRI data were processed with Statistical Parametric Mapping 8 (SPM8) (http://www.fil.ion.ucl.ac.uk/spm) and Resting-State fMRI Data Analysis Toolkit (REST) (http://www.restfmri.net). For each participant, the first 10 volumes of scanned data were deleted due to magnetic saturation effects, then the remaining images were preprocessed using the following steps: slice timing, head motion correction, spatial normalization to the standard Montreal Neurological Institute (MNI) space and resampled into 3×3×3 mm^3^ voxels, followed by spatial smoothing with a Gaussian filter of 6 mm full-width at half-maximum (FWHM). According to the record of head motion within each fMRI run, Participants were excluded if their head motion was>2.5mm maximum displacement in any of the x, y or z directions or 2.5°of any angular motion throughout the course of the scan. Preprocessing in REST consisted of removing linear drift through linear regression and temporal band-pass filtering (0.01–0.08Hz) to reduce the effects of low-frequency drifts and physiological high-frequency noise. To remove the effects of the nuisance covariates, linear regression of head motion parameters, global mean signal, white matter signal and cerebrospinal fluid signal were performed.

#### Definition of ROI

The bilateral hippocampus ROI was defined according to the automated anatomical labeling (AAL) template[39] contained in REST, which has been resampled to 3 ×3 ×3 mm^3^. For each subject, the BOLD time series of the voxels within the ROI were averaged to generate the reference time series for this ROI. A PFC mask was created using the normalized T1-weighted high-resolution images of all participants, which were stripped using BrainSuite2 (http://brainsuite.usc.edu). The PFC mask included 20 labels of AAL template (bilateral SFGdor, ORBsup, MFG, ORBmid, IFGoperc, IFGtriang, ORBinf, SFGmed, ORBsupmed and ACG), corresponding to Brodmann areas (BA) 9–12, 24, 25, 32, and 44–47. Only voxels within this mask were further analyzed[40].

For each subject, the mean time course for the hippocampus ROI was calculated by averaging the time course for all voxels within the hippocampus ROI. Correlation analysis was carried out between the seed ROI and PFC in a voxel-wise manner using REST. The correlation coefficients were then transformed to z-values using the Fisher r-to-z transformation.

### Statistical analysis

Two-sample t tests and χ^2^tests were used to compare demographic data and HAMD scores between the MDD and HC groups (p<0.05). Two sample t-tests were used to compare group differences in FA values in fornix (p<0.05) in SPSS. Another two sample t-test was used to explore differences in functional connectivity between patients and controls in PFC. The contrast map threshold was set at p<0.05 for each voxel, with a cluster size of at least 48 voxels (1296mm^3^), which was equal to the corrected threshold of p<0.05, as determined by AlphaSim (see program AlphaSim by B.D. Ward in AFNI software. http://afni.nimh.nih.gov/pub/dist/doc/manual/AlphaSim.pdf).

To explore the relationship between structural and functional connectivity, we investigated the correlations in MDD and HC participants separately between the FA values of fornix and the strength of the functional connectivity in the PFC regions showing significant differences between the two groups. We used Pearson’s correlation analyses, significant correlation was set at p < 0.05(two-tailed).

We also performed Pearson correlation analyses in MDD adolescents to assess the correlation of HAMD-17 scores with the FA values of fornix and z scores in the PFC regions showing significant differences between the MDD and HC groups. Additionally, Spearman correlation analyses were performed in MDD participants to assess the correlation of illness duration with the FA values of fornix and z scores in the PFC regions that were significantly different between MDD and HC groups.

## Results

### Demographic and Clinical Scales

There were no significant differences in age (p = 0.994), gender (p = 0.155) or education (p = 0.133) between the adolescent MDD and HC groups. Compared to HC, MDD adolescents had significantly greater levels of depression as measured by the HAMD-17 (Table 1).

**Table 1:** Demographic and clinical data of participants.

Characteristic	MDD	HC	Statistic	p-value
Number	26	31		
Age (year, mean±SD)	15.6±1.27.	15.6±1.38	t = 0.007	0.994
Gender (male/female)	7/19	14/17	χ^2^ = 2.02	0.155
Education (year, mean±SD)	9.88±1.34	10.5±1.69	t = -1.53	0.133
HAMD-17 score(mean±SD)	21.35±7.35	1.33±1.65	t = 13.59	<0.001*
Illness duration (month, mean±SD)	7.94±10.49	NA	NA	NA

Notes: SD, standard deviation; MDD, major depression disorder; HC, healthy controls; HAMD, Hamilton Depression Rating Scale; NA, not applicable.

*p<0.05 was considered statistically significant

### DTI and FC Results

Compared with healthy controls, MDD group had significant lower FA values in the fornix (MDD: mean 0.45 [standard deviation (SD) 0.08], HC: mean 0.49 [standard deviation (SD) 0.03], t = 2.471, p = 0.017).

**Fig 1:**
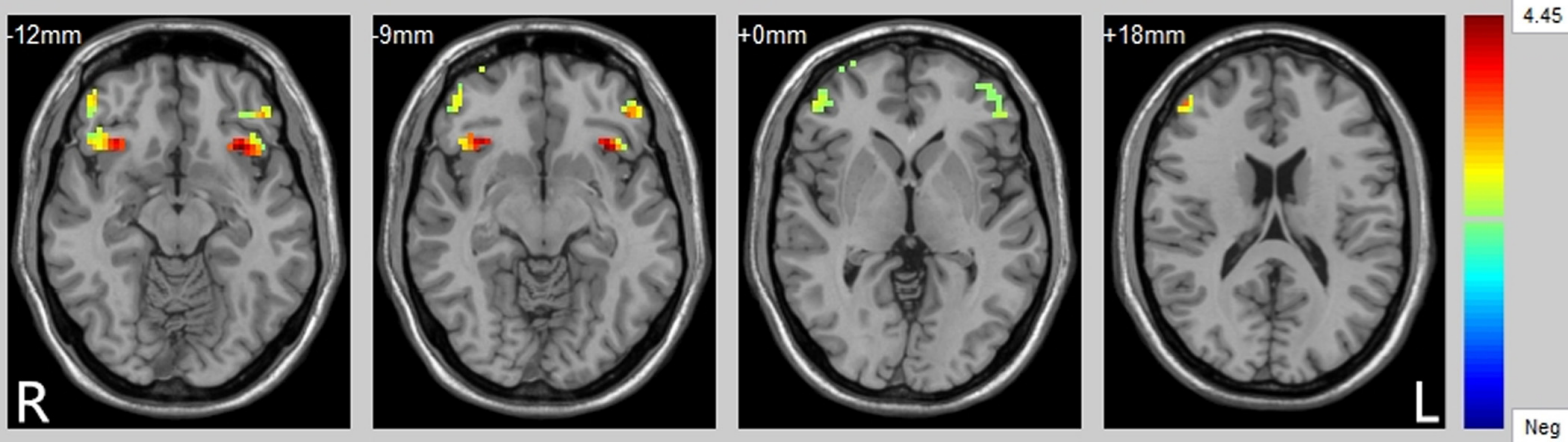
The axial images (MNI coordinate z = –12mm,-9mm,0mm,+18mm) display the regions in bilateral ventral prefrontal cortex (VPFC) and bilateral dorsal lateral prefrontal cortex (DLPFC) that show decreased functional connectivity from the bilateral hippocampus in adolescents with major depressive disorder (MDD), compared to healthy controls (HC) at rest. The color bar represents the range of T values. L, left brain; R, right brain.

We found four significant different PFC regions between healthy control adolescents and those with depression (Table 2; Fig 1). Compared with the HC group, the MDD group showed significant decreased rsFC between the bilateral hippocampal and PFC regions that included left VPFC (BA 47), right VPFC (BA 47), left VPFC and DLPFC (BA 47/10/11/46), right VPFC and DLPFC (BA 10/46/11/47/45) (Table 2; Fig 1). These findings correspond to a corrected p <0.05 by AlphaSim correction.

**Table 2:** Brain regions showing significant differences in functional connectivity of the bilateral hippocampal-ROI between groups.

Brain regions	Cluster size	BA	MNI Coordinates			T Values
	voxels		x	y	z	(peak)
Left VPFC	56	47	-33	21	-12	4.45
Right VPFC	49	47	33	24	-9	4.02
Left VPFC/DLPFC	63	47/10/11/46	-45	39	-9	3.45
Right VPFC/DLPFC	89	10/46/11/47/45	48	45	18	3.35

Notes: VPFC, ventral prefrontal cortex; DLPFC, dorsal lateral prefrontal cortex; BA, Brodmann area.

Furthermore, significant negative correlations were observed between fornix FA values and functional connectivity from hippocampus to left VPFC and DLPFC (BA 47/10/11/46) (r = -0.512; p = 0.009) within the control group (Table 3). However, there was no significant correlation between the fornix FA and the strength of functional connectivity within the patient group.

**Table 3:** The correlation between the strength of these functional connectivities and the FA Value of the fornix within each group.

Brain regions	FA within patients		FA within controls	
	r	P	r	P
Left VPFC	0.12	0.559	-0.023	0.913
Right VPFC	-0.129	0.215	-0.257	0.53
Left VPFC/DLPFC	0.05	0.807	-0.512	0.009*
Right VPFC/DLPFC	-0.165	0.419	-0.109	0.603

Notes: VPFC, ventral prefrontal cortex; DLPFC, dorsal lateral prefrontal cortex.

*p<0.05 was considered statistically significant

Neither the HAMD scores nor the illness duration had any significant associations with FA values in the fornix and PFC functional connectivity in MDD adolescences.

## Discussion

In this study, we found decreased FA values in the fornix in adolescents with MDD, as well as decreased hippocampus-PFC rsFC, compared to HC. Furthermore, we identified the association of fornix white matter integrity and PFC-hippocampus functional connectivity in HC; however, these associations were disappeared in MDD adolescence. To our knowledge, this is the first study combining DTI and rs-fMRI methods to investigate the PFC-hippocampus neural circuitry and the relationship between structural and functional connectivity in this circuitry in the adolescents with MDD. Our findings implicated the dysfunction of PFC-hippocampus neural circuitry in the early onset of depression.

The fornix is the major output fiber tract for the hippocampus, projecting to mammillary bodies, anterior nuclei of thalamus and PFC. Reduced fornix FA values have been detected in several mental disorders such as schizophrenia[41], bipolar disorder[42] and Alzheimer’s disease[43] and associated with decreased hippocampus volume[44]. Convergent studies of MDD have demonstrated reduced hippocampus volume in adults[15,45] as well as adolescents[46,47]. In a recent DTI study, Li et al reported white matter disruptions in the fornix and hippocampal cingulum in late-life depression[48]. As the first study in medication-naïve adolescents MDD, the current findings of altered FA values in the fornix provided primary evidence that white matter abnormalities in PFC-hippocampus circuitry may be present in the early stages of MDD and play an important role in the pathophysiology of adolescent MDD.

In this study, we also demonstrated decreased rsFC between the hippocampus and VPFC/DLPFC in adolescent MDD compared with healthy controls. The hippocampus has been proven to play an important role in memory[49] and emotion processing[50]. Functional abnormalities of the hippocampus in adult MDD have been consistently reported in several fMRI studies[51,52,53,54]. The DLPFC is one of key regions involving in mood regulation and cognitive functioning which are frequently implicated in the pathophysiology of depression[6], while the VPFC is involved in reward processing and related to reduced enjoyment in depression[55]. Abnormal DLPFC and VPFC activation within adult MDD have also been detected in both facial recognition[56,57] and cognitive control[58]. In reward processing fMRI studies, Frobes reported that depressed adolescents had blunted response in the VPFC, which may cause an inadequately emotional response to reward[59,60]. Taken together, our findings of decreased PFC-hippocampus rsFC may reflect emotional and cognitive dysfunction in adolescent MDD. Functional disconnectivity in PFC-hippocampus circuitry may the early sign of adult MDD.

As human neuroimaging studies suggest that brain structural and functional networks may share similar pathways[61,62], interesting questions were raised on the association between structural and functional connectivity in MDD. Until now, few studies detected the relationship between structural and functional connectivities in MDD. Steffens et al found relationship between left VPFC-temporal regions (including amygdala and hippocampus) rsFC and left uncinate fasciculus FA in geriatric depression[63]; de Kwaasteniet et al reported that adult MDD showed higher subgenual ACC-hippocampus functional connectivity and decreased uncinate fasciculus FA[36], suggesting that structural abnormalities may contribute to disrupted functional connectivity in PFC-hippocampus circuitry. To our knowledge, this is the first study using multiple MRI techniques to investigate structural and functional connectivity in adolescent MDD. Our findings of decreased rsFC between hippocampus and PFC and decreased FA of fornix which links the two regions within adolescent MDD provide the primary evidence of structural and functional abnormalities in the early stage of MDD. Furthermore, significant negative correlations were observed between fornix FA values and the strength of PFC-hippocampus functional connectivity within the control group. However, there was no significant correlation between the fornix FA and the strength of functional connectivity within the patient group. These results are similar with our previous findings in schizophrenia[64]. We speculate that the negative corralations between the strength of structural and functional connectivity may reflect the compensatory mechanism that enhanced functional connectivity could complement lower structural connectivity to maintian the balance of PFC-hippocampus circuitry in HC. Abnormalities of both structural and functional connectivies may damage the compensatory mechanism and result in the imbalance of PFC-hippocampus circuitry in MDD. Hence disrupted structural-functional relationship may play an specific role in the neuropathophysiology of MDD and need to be further investigated in future.

Some limitations of this study should be noted. First, as we select first-episode medication-naïve adolescent MDD to minimize the confounds of chronicity, treatment or comorbidity, the relative small sample size may limit the generalizability of our results as well as our ability to detect relationships between clinical variables and neuroimaging findings in this study. Secondly, since we did not detect significant correlation between the white matter integrity and the strength of functional connectivity in adolescent MDD, the direct relation between structural and functional connectivity within PFC-hippocampus neural circuitry is still unclear. We speculate that the relative small sample size may limit our ability to detect the relation as well as the generalization of our results. Future studies with large sample size is important to further understand the neuropathophysiology of MDD. Furthermore, the interpretation of results should be cautious because follow-up studies have found that 20 to 40% of adolescents with MDD develop BD within a period of 5 years after the onset of depression[65]. The cross-sectional design of this study did not allow us to distinguish between trait MDD subjects and those converting to bipolar disorder in future, longitudinal studies are needed to examine the difference between them.

In summary, our study of first-episode medication-naïve adolescent MDD demonstrated decreased structural and functional connectivity as well as deficits of the association between structural and functional connectivity shown in HC in the PFC-hippocampus neural circuitry. These findings suggest that abnormal PFC-hippocampus neural circuitry may present in the early onset of MDD and play an important role in the neuropathophysiology of MDD.
